# Neonatal hyperbilirubinemia: Assessing variation in knowledge and practice

**DOI:** 10.1371/journal.pone.0282413

**Published:** 2023-02-28

**Authors:** Danielle Owerko, Kelsey Ryan, Erwin Cabacungan, Ke Yan, Kris Saudek

**Affiliations:** 1 Division of Hospital Medicine, Department of Pediatrics, Medical College of Wisconsin, Milwaukee, Wisconsin, United States of America; 2 Division of Neonatology, Department of Pediatrics, Medical College of Wisconsin, Milwaukee, Wisconsin, United States of America; 3 Division of Quantitative Health Sciences, Department of Pediatrics, Medical College of Wisconsin, Milwaukee, Wisconsin, United States of America; St Paul’s Hospital Millennium Medical College, ETHIOPIA

## Abstract

**Introduction:**

Neonatal hyperbilirubinemia (NH) is commonly diagnosed and managed by pediatricians in various clinical settings. The 2004 American Academy of Pediatrics (AAP) Clinical Practice Guideline on NH is widely cited, but literature examining variation across pediatric specialties is limited. This study aimed to assess baseline knowledge and practice habits regarding NH among pediatric providers across various specialties immediately prior to the release of the 2022 NH clinical practice guideline.

**Methods:**

A non-probability, convenience, self-selected sampling survey was electronically distributed to 311 subjects across five specialties within one pediatric healthcare institution. The survey included eight multiple choice knowledge-based questions with confidence assessments and five management-based questions assessing respondent agreement on a 5-point scale. To compare groups, the Kruskal-Wallis and Mann-Whitney tests were used for continuous variables, and the chi-square and Fisher’s exact tests were used for categorical variables.

**Results:**

The overall survey response rate is 46%. There were significant differences between specialties’ knowledge regarding NH (p<0.05). There were also significant differences between specialties’ confidence ratings, independent of choosing the correct response (p<0.05). For select management-based questions, there were also significant differences between specialties (p<0.05). A majority of respondents (56%) indicated phototherapy treatment thresholds should remain the same in updated management guidelines.

**Conclusions:**

Significant variations in knowledge and management of NH were identified among pediatric specialties. This suggests dissemination of new guidelines must be cognizant of different constraints impacting knowledge and practice across specialties.

## Introduction

Pediatricians commonly diagnose and manage neonatal hyperbilirubinemia (NH) in various healthcare settings [1–6]. Defined as an elevated total serum bilirubin (TSB) level, hyperbilirubinemia clinically manifests as jaundice and affects a majority of all newborn infants [4, 5]. The most severe risk of untreated hyperbilirubinemia is kernicterus, characterized by long-term neurologic damage from bilirubin deposits in the brain [5, 6]. In 2004, with an update in 2009, the American Academy of Pediatrics (AAP) published a Clinical Practice Guideline (CPG) entitled Management of Hyperbilirubinemia in the Newborn Infant 35 or More Weeks of Gestation [1]. This CPG has since served as the main evidence-based set of recommendations guiding management of NH across clinical environments. At the time this survey was conducted, our institution used the 2009 recommendations, including when and where to initiate phototherapy, as the basis for our local practice pathway that is referenced when caring for infants in the newborn nursery, acute care floor, neonatal intensive care unit (NICU), emergency department (ED), and outpatient primary care practices.

In August 2022, the AAP published a revised CPG which updated the common risk factors for severe NH and adjusted phototherapy thresholds across all gestational age categories based on extensive review of new evidence [2, 3]. It is anticipated that providers will promptly begin using the new guidelines to inform their clinical decision-making regarding NH.

A review of existing literature identified studies that examined providers’ knowledge and practice habits within individual specialties [7, 8], or amongst a select few primary care specialties [9], but none have studied where variations exist between all pediatric specialties that most commonly manage the care of jaundiced infants. Therefore, the primary aim of this study was to assess baseline knowledge and current clinical practice habits amongst all specialties that manage NH within a single healthcare institution.

## Methods

A non-probability, convenience, self-selected sampling survey was created by the authors using Qualtrics [10]. A comprehensive literature review regarding NH was conducted and used to guide question development. The survey consisted of two demographic questions and eight multiple choice knowledge-based questions with corresponding confidence assessments for select questions (sliding scale 0–100). We also assessed respondent agreement with five NH management scenarios (“practice habits”) on a 5-point scale. Full text of the survey, with citations and correct answers highlighted, can be found in S1 File.

The preliminary survey was shared with a group of subject matter experts and learners who provided feedback regarding question content and time to complete the survey. Once finalized, the survey was electronically disseminated to 311 physicians at our free-standing tertiary care children’s hospital in the Midwest United States. Invited respondents were pediatric hospitalists, neonatologists, pediatric emergency medicine (PEM) physicians, pediatric residents, and outpatient primary care pediatricians. The survey was first disseminated on October 11, 2021 and two reminder emails were subsequently sent before the survey closed on November 12, 2021. An informed consent process utilizing an informational letter was approved by the Institutional Review Board of the Medical College of Wisconsin. Survey recipients were explicitly advised of their prerogative to not answer individual questions or the survey as a whole to safeguard respondent autonomy.

Data were extracted from Qualtrics and analyzed using SAS [11]. Surveys with missing demographic responses or no completed knowledge and practice questions were excluded from analysis.

To compare groups, the Kruskal-Wallis and Mann-Whitney tests were used for continuous variables, and the Chi-square and Fisher’s exact tests were used for categorical variables.

## Results

**Fig 1:**
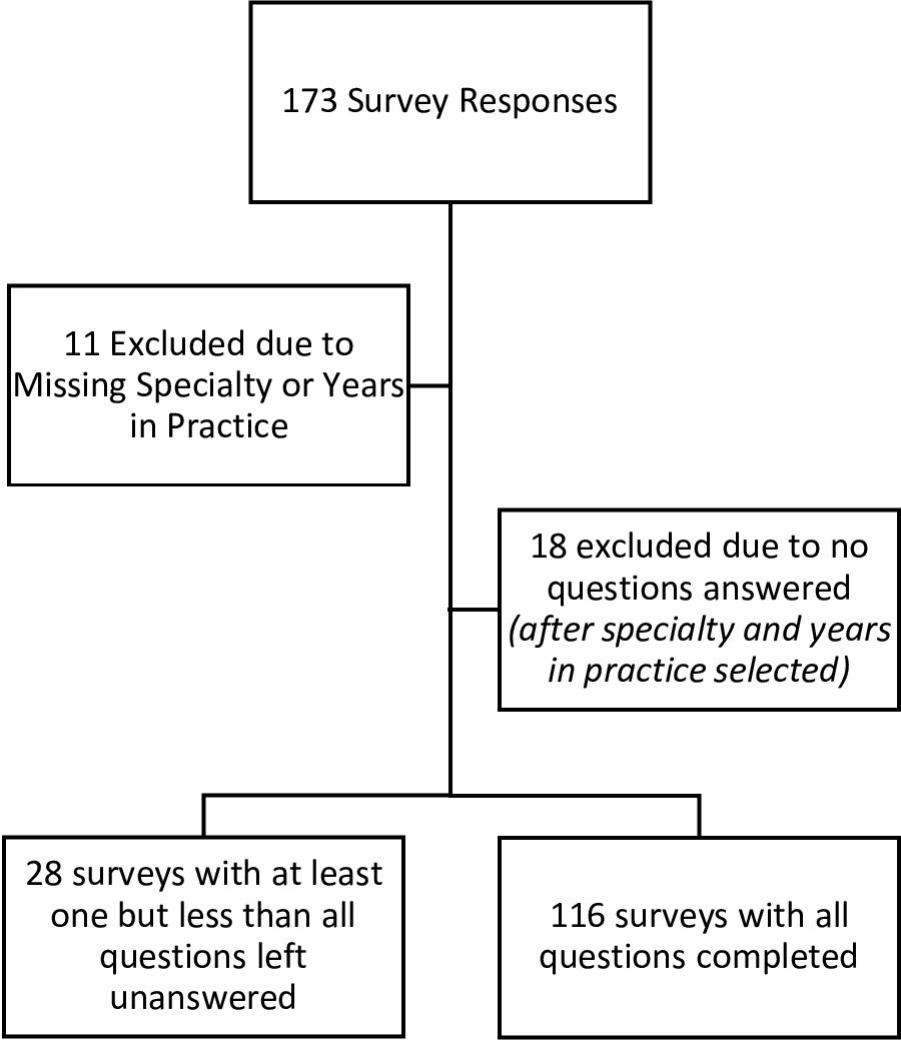
Included and excluded survey responses.

We received 173 individual survey responses, and 144 responses were ultimately analyzed for an overall response rate of 46% (144/311). Response rates by specialty were hospital medicine 64% (25/39), PEM 50% (16/32), NICU 61% (14/23), primary care 50% (56/113) and pediatric residents 32% (33/104). Survey inclusion and exclusion are summarized in Fig 1.

Survey respondents were first asked to identify their specialty (D1) and years in practice (D2). Specialty and number of years in practice were highly correlated (p<0.0001). For this reason, this analysis reports trends overall and by specialty subgroup, and not in both specialty and years in practice subgroups.

**Fig 2:**
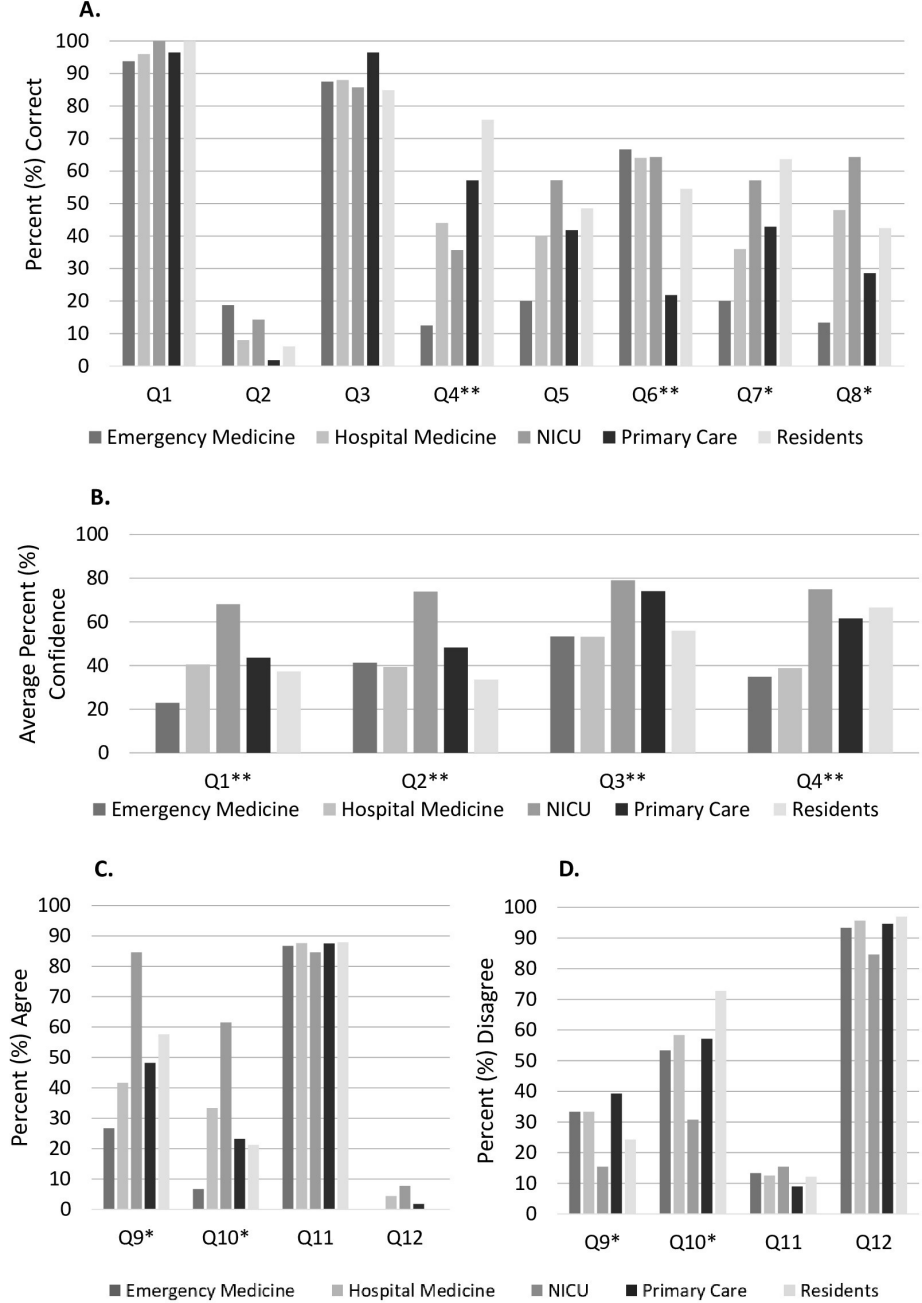
Graphical representation of survey responses by specialty of respondent. **A.** Percent Correct Response Rate by Specialty for Knowledge Questions. **B.** Average Self-Reported Percent Confidence by Specialty. **C.** Percent Agree [Strongly Agree + Somewhat Agree] with Described Management by Specialty. **D.** Percent Disagree [Strongly Disagree + Somewhat Disagree with Described Management by Specialty. *denotes p<0.*05*, **denotes p< 0.*001*.

Table 1 presents the survey questions and statistically significant differences in responses by specialty. Differential responses by specialty are further illustrated in Fig 2. Of the eight baseline knowledge questions, only three questions were answered correctly by a majority of respondents (Q1, 3 & 4). Correct response rates by specialty are illustrated in Fig 2A.

**Table 1:** Survey questions, responses, and differences by specialty.

Question Number	Survey Question and Response^1^			Differences by specialty^2^	
*Knowledge Questions*		*Percent Correct or Affirmed*	*Number Missing*	*Percent Correct*	*Confidence (Rated 1–100)*
Q1	What is the normal physiologic rate of rise for bilirubin in a term infant who is feeding well?	97.2%	0	p = 0.57	p = 0.0004*
Q2	For a term infant with physiologic jaundice who is feeding well, bilirubin most commonly peaks at what level?	7%	1	p = 0.069	p<0.0001*
Q3	For a term infant with physiologic jaundice who is feeding well, bilirubin most commonly peaks at what day of life?	90.3%	0	p = 0.23	p = 0.0008*
Q4	Transcutaneous bilirubin underestimates, matches, overestimates or does not correlate with total serum bilirubin level?	52.1%	0	p = 0.0005*	p<0.0001*
Q5	A cord bilirubin <3mg/dl is reassuring in a Coombs positive term infant with ABO incompatibility. (Agree, Neutral, Disagree)	Agree: 42.2%	2	p = 0.3	
Q6	ABO incompatibility is not as severe as Rh incompatibility. (Agree, Neutral, Disagree)	Agree: 45.8%	2	p = 0.0002*	
Q7	Prenatal maternal Rhogam administration can cause neonates to have a positive Coombs result. (Agree, Neutral, Disagree)	Agree: 45.5%	1	p = 0.038*	
Q8	There are long term risks associated with phototherapy. (Agree, Neutral, Disagree)	Agree: 37.1%	1	p = 0.022*	
*Practice Habits*		*Percent Affirm* ^3^	*Number Missing*	*Five Point Scale of Agreement* ^4^	*Three Point Scale of Agreement* ^5^
Q9	I am comfortable relying on transcutaneous bilirubin measurements for monitoring of neonatal jaundice.	50.4%	3	p = 0.11	p = 0.048*
Q10	Every term newborn should have a DIRECT bilirubin measurement prior to discharge from the newborn nursery.	26.2%	3	p = 0.047*	p = 0.01*
Q11	A well-appearing, full term, exclusively breastfed newborn has a high intermediate risk bilirubin level prior to discharge from the newborn nursery. The infant is sent home with clinic follow up arranged within 24 hours. Please rate your agreement with this management.	87.2%	3	p = 0.4	p = 0.95
Q12	I would recommend hospital admission and initiation of phototherapy for a well-appearing term infant whose bilirubin level is 2-3mg/dl below phototherapy threshold.	2.1%	4	p = 0.031*	p = 0.49
Q13	Revised AAP Phototherapy Guidelines are anticipated. In your opinion, for a term infant with no risk factors, phototherapy thresholds should:		3	p = 0.023*	
	Initiate treatment at lower bilirubin levels than they currently do	(1) 2.1%			
	Remain the same	(2) 56%			
	Initiate treatment at higher bilirubin levels than they currently do	(3) 41.8%			

^1^ Survey text with correct answers highlighted can be found in S1 File

^2^ Kruskal-Wallis test and Mann-Whitney test were used for continuous variables, and the Chi-square test and the Fisher’s exact test were used for categorical variables.

^3^ “Percent Affirm” reports percentage of survey responses that indicate “strongly agree” or “somewhat agree”

^4^Five category scale of agreement: “strongly agree”, “somewhat agree”, “neither agree nor disagree”, “somewhat disagree”, “strongly disagree”

^5^Three category scale of agreement: [“strongly agree”+”somewhat agree”], [“neither agree nor disagree”], [“somewhat disagree”+ “strongly disagree”]

* A p-value <0.05 is considered statistically significant.

Knowledge questions Q1-4 were accompanied by a confidence assessment with responses by specialty illustrated in Fig 2B. There was a significant difference in confidence levels by specialty for all confidence assessments, with NICU consistently more confident in their responses than other specialties. This pattern of confidence persisted independently of identifying the correct answer. For example, there were significant differences in how specialties answered question 4. Residents selected the correct answer more frequently than NICU, hospital medicine, and PEM. NICU remained more confident in their answer than primary care, hospital medicine, and PEM, but not residents.

Respondents differed in their answers to practice habits questions by specialty, except in Q11, as demonstrated in Fig 2C and 2D). In Q11, the majority of respondents (87.2%) reported that they agreed with outpatient follow-up within 24 hours for high intermediate risk bilirubin level prior to discharge from the newborn nursery, regardless of specialty of the respondent. Of note, in Q13, 56% of respondents indicated that they were not in favor of modifying phototherapy thresholds. PEM and primary care more frequently responded this way in comparison to hospital medicine providers and residents. PEM and primary care were also the only specialties where respondents reported they would be in favor of initiating treatment at lower bilirubin levels than the 2004 guidelines indicate.

Missing data frequencies by survey question are included in Table 2. There was no significant difference between specialties or years in practice within groups who answered none or less than all survey questions. Data analysis proceeded, assuming missing data occurred completely at random.

**Table 2:** Missing data by survey question. ‘X’ indicates non-missing. ‘O’ or ‘.’ are missing.

Q1	Q1a_1	Q2	Q2a_1	Q3	Q3a_1	Q4	Q4a_1	Q5	Q6	Q7	Q8	Q9	Q10	Q11	Q12	Q13	Freq	%
X	X	X	X	X	X	X	X	X	X	X	X	X	X	X	X	X	116	71.60
X	X	X	X	X	X	X	X	X	X	X	X	X	X	X	.	X	1	0.62
X	X	X	X	X	X	X	X	X	X	X	X	.	.	.	.	.	2	1.23
X	X	X	X	X	X	X	X	.	X	X	X	X	X	X	X	X	1	0.62
X	X	X	X	X	X	X	X	.	.	.	.	.	.	.	.	.	1	0.62
X	X	X	X	X	.	X	X	X	X	X	X	X	X	X	X	X	3	1.85
X	X	X	X	X	.	X	X	X	.	X	X	X	X	X	X	X	1	0.62
X	X	X	.	X	X	X	X	X	X	X	X	X	X	X	X	X	1	0.62
X	.	X	X	X	X	X	X	X	X	X	X	X	X	X	X	X	9	5.56
X	.	X	.	X	X	X	X	X	X	X	X	X	X	X	X	X	3	1.85
X	.	X	.	X	.	X	.	X	X	X	X	X	X	X	X	X	5	3.09
X	.	.	.	X	X	X	X	X	X	X	X	X	X	X	X	X	1	0.62
O	O	O	O	O	O	O	O	O	O	O	O	O	O	O	O	O	18	11.11

## Discussion

Our survey assessed baseline knowledge and current practice habits regarding NH amongst pediatric providers across various specialties and clinical settings within one hospital system. We found significant variations in knowledge, practice habits, and confidence between specialties. A majority of respondents also indicated that phototherapy treatment thresholds should remain the same in future management guidelines. Our results underscore the need for thoughtful implementation of the newly released 2022 AAP neonatal hyperbilirubinemia CPG [2].

Clinical practice guidelines are intended to disseminate up-to-date research and improve value-based care. Variation in care despite published CPGs is well documented in the literature [12–17]. Noncompliance with clinical practice guidelines is reported to be as high as 70% [13] and occurs in academic teaching hospitals, where guidelines often originate, and in all disciplines of medicine [12, 15–17]. Barriers to adopting CPGs include lack of awareness or agreement, static practice and limited motivation to change, and external factors such as lack of time [13]. Importantly, when CPGs are implemented successfully, they improve patient outcomes [18–20].

Our survey incorporated questions about basic bilirubin physiology [3–6] in addition to management that was articulated in the 2004 and 2009 updated AAP Clinical Practice Guidelines on NH [1]. Our results reflect suboptimal knowledge and compliance with the guidelines that were published almost twenty years ago. This suggests that CPGs do not always result in changes in patient care. A landmark report published by the Institute of Medicine in 2001, Crossing the Quality Chasm: A New Healthcare System for the 21^st^ Century, called for adoption of evidence-based practice to improve patient outcomes [21]. It is clear we need to rethink how we answer this call.

It may be difficult for providers to adapt to new standards of clinical care without updating their underlying medical knowledge. Deficits in knowledge occurred across specialties in our survey data, but our pediatric residents, with the least years in practice, were tied for the most overall correct responses. We speculate this reflects the commitment residents demonstrate to learning while in training. As articulated by the Accreditation Council for Graduate Medical Education, lifelong learning is a core competency of our profession [22].

Our study has several limitations. First, this was a single-center study, so results may not be generalizable to other institutions. It can, however, be informative for individual hospital systems to understand their own patterns of variability when operationalizing new guidelines, and we present one strategy to accomplish this. Second, we could not distinguish between the effects of specialty and years in practice when analyzing our survey results. The close relationship between specialty and years in practice is logical because different specialties require different years in practice to attain board certification. This is an opportunity for further study, potentially with a larger sample size. Third, our survey instructions asked that participants not use outside resources while ensuring that they would not be graded or judged based on their answers. We acknowledge that some providers may have felt uncomfortable encountering questions they did not know how to answer. We also cannot guarantee that providers did not use outside resources when responding, which would increase the percentage of correct responses. Lastly, our overall response rate was less than 50%, potentially reflecting selection bias for confident respondents.

Our survey results suggest that barriers to compliance with well-established guidelines in the management of NH exist in our institution. Additionally, these barriers may differ across provider groups who regularly manage infants with jaundice. This suggests that the dissemination of the new guidelines must be cognizant of differing constraints on knowledge and practice across specialties and adaptable across practice settings. At our institution, these results suggest a need for faculty development resources designed with multidisciplinary input and which address NH pathophysiology as well as recommended changes to management. Implementation of the 2022 CPG presents an opportunity for pediatric healthcare organizations like ours to align practice and reduce variation in care across specialties that manage NH.

## Conclusion

Significant variations in knowledge and management of NH were identified among pediatric specialties at our institution. This suggests dissemination of new guidelines must be cognizant of different constraints impacting knowledge and practice across specialties.

## Supporting information

S1 File Survey text with correct answers and associated references. (DOCX)
