# Supplementary material for: Neonatal hyperbilirubinemia: Assessing variation in knowledge and practice
Source: PLoS One. 2023 Feb 28;18(2):e0282413. doi: 10.1371/journal.pone.0282413 (PMC9974112; doi:10.1371/journal.pone.0282413)
Supplement: S1 File — (DOCX) [file pone.0282413.s001.docx]

**Supplement 1.** Survey text with correct answers and associated references

**Bilirubin in the Newborn: Knowledge & Practice Habits**

Neonatal hyperbilirubinemia is commonly diagnosed ad managed by pediatricians in various healthcare settings. A new and updated clinical practice guidelines is anticipated from the American Academy of Pediatrics (AAP) in the near future. The aim of this study is to assess knowledge regarding bilirubin in the newborn and gather information about current practice habits amongst the providers in our community.

You are not being graded or judged based on whether you answer a question correctly or not. **Please answer without the use of outside sources or discussions with other providers.**

**D1** Please identify your specialty of practice:

- Primary care
- Hospital Medicine
- NICU
- Emergency Medicine
- Resident PL-1
- Resident PL-2
- Resident PL-3 or above

**D2** Years in practice:

- 0-10 years
- 11-20 years
- 21-30 years
- 30+ years

**Q1** What is the normal physiologic rate of rise for bilirubin in a term infant who is feeding well?

- **0.05 mg/dL/hr**
- **0.1 mg/dL/hr**
- **0.2 mg/dL/hr^1^**
- 0.5 mg/dL/hr
- 1.0 mg/dL/hr

**Q1a** Please rate how confident you are in your answer to Question 1 above:

- Not at all confident [sliding scale 1-100] Extremely confident

**Q2** For a term infant with physiologic jaundice who is feeding well, bilirubin most commonly peaks at what level?

- 3-5 mg/dL
- **5-6 mg/dL^1^**
- 7-9 mg/dL
- 10-12 mg/dL
- 13-15 mg/dL

**Q2a** Please rate how confident you are in your answer to Question 2 above:

- Not at all confident [sliding scale 1-100] Extremely confident

**Q3** For a term infant with physiologic jaundice who is feeding well, bilirubin most commonly peaks at what day of life?

- DOL 0-2
- **DOL 3-5^1^**
- DOL 6-8
- DOL 9-11
- DOL 12-14

**Q3a** Please rate how confident you are in your answer to Question 3 above:

- Not at all confident [sliding scale 1-100] Extremely confident

**Q4** Transcutaneous bilirubin ______________ the total serum bilirubin level.

- Underestimates
- Matches
- **Overestimates^2,3^**
- Does not correlate with

**Q4a** Please rate how confident you are in your answer to Question 4 above:

- Not at all confident [sliding scale 1-100] Extremely confident

**Q5** A cord bilirubin <3 mg/dL is reassuring in a Coombs positive infant with ABO incompatibility.

- **Agree^4^**
- Neutral
- Disagree

**Q6** ABO incompatibility is not as severe as Rh incompatibility.

- **Agree^5^**
- Neutral
- Disagree

**Q7** Pre-natal maternal Rhogam administration can cause neonates to have a positive Coombs result.

- **Agree^6^**
- Neutral
- Disagree

**Q8** There are long term risk associated with phototherapy.

- **Agree^7-9^**
- Neutral
- Disagree

**Q9** I am comfortable relying on transcutaneous bilirubin measurements for monitoring of neonatal jaundice.

- Strongly agree
- Somewhat agree
- Neither agree nor disagree
- Somewhat disagree
- Strongly disagree

**Q10** Every term newborn should have a DIRECT bilirubin measurement prior to discharge from the newborn nursery.

- Strongly agree
- Somewhat agree
- Neither agree nor disagree
- Somewhat disagree
- Strongly disagree

**Q11** A well-appearing, full term, exclusively breastfed newborn has a high intermediate risk bilirubin level prior to discharge from the newborn nursery. The infant is sent home with clinic follow up arranged within 24 hours. Please rate your agreement with this management.

- Strongly agree
- Somewhat agree
- Neither agree nor disagree
- Somewhat disagree
- Strongly disagree

**Q12** I would recommend hospital admission and initiation of phototherapy for a well appearing, term infant whose bilirubin level is 2-3 mg/dL below phototherapy threshold.

- Strongly agree
- Somewhat agree
- Neither agree nor disagree
- Somewhat disagree
- Strongly disagree

**Q13** Revised AAP Phototherapy Guidelines are anticipated. In your opinion, for a term infant with no risk factors, phototherapy thresholds should:

- Initiate treatment at lower bilirubin levels than they currently do
- Remain the same
- Initiate treatment at higher bilirubin levels than they currently do

**Survey References:**

1. Shaughnessy E, Goyal N. Digestive System Disorders. In: Kliegman R, Geme JS, Blum N, Shah S, eds. Nelson Textbook of Pediatrics. Elsevier; 2020:949-961:chap 123.

2. Maisels MJ, Ostrea EM, Jr., Touch S, et al. Evaluation of a new transcutaneous bilirubinometer. Pediatrics. Jun 2004;113(6):1628-35. doi:10.1542/peds.113.6.1628

3. Taylor JA, Burgos AE, Flaherman V, et al. Discrepancies between transcutaneous and serum bilirubin measurements. Pediatrics. Feb 2015;135(2):224-31. doi:10.1542/peds.2014-1919

4. Jones KDJ, Grossman SE, Kumaranayakam D, Rao A, Fegan G, Aladangady N. Umbilical cord bilirubin as a predictor of neonatal jaundice: a retrospective cohort study. BMC Pediatr. Sep 20 2017;17(1):186. doi:10.1186/s12887-017-0938-1

5. Pan DH, Rivas Y. Jaundice: Newborn to Age 2 Months. Pediatr Rev. Nov 2017;38(11):499-510. doi:10.1542/pir.2015-0132

6. Maayan-Metzger A, Schwartz T, Sulkes J, Merlob P. Maternal anti-D prophylaxis during pregnancy does not cause neonatal haemolysis. Arch Dis Child Fetal Neonatal Ed. Jan 2001;84(1):F60-2. doi:10.1136/fn.84.1.f60

7. Maimburg RD, Olsen J, Sun Y. Neonatal hyperbilirubinemia and the risk of febrile seizures and childhood epilepsy. Epilepsy Res. Aug 2016;124:67-72. doi:10.1016/j.eplepsyres.2016.05.004

8. Newman TB, Wu YW, Kuzniewicz MW, Grimes BA, McCulloch CE. Childhood Seizures After Phototherapy. Pediatrics. Oct 2018;142(4)doi:10.1542/peds.2018-0648

9. van der Schoor LWE, van Faassen M, Kema I, et al. Blue LED phototherapy in preterm infants: effects on an oxidative marker of DNA damage. Arch Dis Child Fetal Neonatal Ed. Nov 2020;105(6):628-633. doi:10.1136/archdischild-2019-317024
